# Differences in Kinematic and Kinetic Patterns According to the Bone Tumor Location after Endoprosthetic Knee Replacement Following Bone Tumor Resection: A Comparative Gait Analysis between Distal Femur and Proximal Tibia

**DOI:** 10.3390/jcm10184100

**Published:** 2021-09-10

**Authors:** Sungmin Kim, Changhyun Ryu, Sung-Taek Jung

**Affiliations:** Department of Orthopedic Surgery, Chonnam National University Medical School and Hospital, 42 Jebong-ro, Dong-gu, Gwangju 61469, Korea; kimsum83@gmail.com (S.K.); ryucamel@gmail.com (C.R.)

**Keywords:** bone tumor, knee endoprosthesis, kinematics, kinetics

## Abstract

Modular endoprostheses are frequently used to reconstruct skeletal and knee defects from bone tumor resection and preserve joint function in patients with bone tumors around the knee. Depending on the tumor location, the muscles and extent of the tumor can vary, which can affect gait function. This study aimed to analyze kinetic and kinematic characteristics according to tumor location in patients with endoprosthetic knee replacements after bone tumor resection. Gait analyses were performed in 16 patients who underwent knee endoprosthesis due to tumors around the knee. We divided the patients into distal femur (*n* = 7) and proximal tibia (*n* = 9) groups and conducted between-group comparisons and comparisons with healthy participants. Compared with the control group, the distal femur group showed a tendency for knee extension, and the proximal tibia group showed increased maximal dorsiflexion during stance. The proximal tibia group maintained a flexed hip during the entire gait cycle, compared with the distal femur group. In summary, our results suggest a difference in gait between the distal femur and proximal tibia groups. Patients who have undergone knee prosthesis after knee tumor resection may require different rehabilitation strategies according to the tumor location.

## 1. Introduction

Limb salvage surgery is the standard treatment modality for most malignant or locally aggressive bone tumors, of which approximately 50% arise around the knee [1]. Advances in diagnostic imaging, chemotherapy, and operative techniques have increased the use of limb salvage procedures for patients with bone tumors [2,3]. Modular endoprostheses are frequently used to reconstruct defects after resecting a bone tumor and preserve joint function in patients with tumors around the knee joint [4,5].

Primary bone tumors mainly arise in the distal femur and proximal tibia. The introduction of a rotating hinge design has improved implant survival in endoprosthetic replacement of the distal femur [6]. In addition, the outcomes of endoprosthesis are worse in proximal tibia reconstruction than in distal femur reconstruction [6,7,8]. However, the results for the distal femur cannot be simply compared to the those for the proximal tibia because there are clear differences in both functional outcomes and obstacles in reconstruction [9]. The differences in survival for prosthetic replacements according to tumor location are most likely due to differences in surgical procedure and soft tissue reconstruction. Compared to reconstruction of the distal femur, endoprosthesis reconstruction of the proximal tibia involves inevitable significant muscle loss, and disruption of the extensor mechanism is a major challenge [10,11,12,13,14,15]. The region extensor mechanism is crucial for rehabilitation in patients who undergo reconstruction of the proximal tibia.

Gait function is one of the most significant components of functional outcome evaluation in patients with knee endoprostheses for lower extremity tumors. Previous studies have reported slower walking speed [16,17,18,19], longer step length of the non-operated limb [20], and decreased foot pressure [21] due to insufficient muscle strength around the reconstructed knee. However, these studies did not perform gait analysis according to lesion location, that is, the distal femur or proximal tibia. Thus, this study aimed to determine the kinetic and kinematic characteristics of patients who underwent endoprosthetic knee replacement for tumors around the knee and compare them according to tumor location. We hypothesized that there could be a difference in gait pattern depending on tumor location (i.e., distal femur or proximal tibia).

## 2. Methods

### 2.1. Study Design and Patients

This retrospective study was approved by the institutional review board of our hospital. The subjects were patients treated for bone tumors around the knee joint between January 2001 and January 2018. Patients aged >15 years who underwent endoprosthetic knee replacement after bone tumor resection, and who had no neurological or musculoskeletal pathology that affected gait function, were eligible. The exclusion criteria were concurrent metastasis, local recurrence, history of revision surgery, unstable implant, <2 years since surgery, daily use of walking aid or orthopedic shoes, >10° varus or valgus deformity at the knee joint, and >2.5 cm discrepancy in limb length. All patients underwent reconstruction using a single modular universal tumor and revision system (MUTARS; Implantcast GmbH, Buxtehude, Germany). The patients were divided into the distal femur group (DFG) and the proximal tibia group (PTG) according to bone tumor location, and between-group comparisons in gait were conducted.

Of the 23 eligible patients, 7 were excluded because of a history of revision surgery (*n* = 2), tumor location in both distal femur and proximal tibia (*n* = 2), implant instability (*n* = 2), and <2 years since the last surgery (*n* = 1). Finally, 16 patients (6 women and 10 men) were included in this study (Table 1). The mean age at the time of surgery was 22.10 years (range, 14.5–49.58); 8 tumors involved the left side, and 8 the right side. The patients were 5.3 years (range, 2.0–14.0) post-surgery at follow-up. With respect to tumor diagnosis, 15 patients had osteosarcoma, 2 patients had giant cell tumors, and 1 patient had Ewing’s sarcoma. The DFG included 7 patients, and the PTG included 9 patients.

Moreover, gait was compared with that of a control group consisting of 18 healthy participants. All healthy control participants did not have orthopedic, neurological, respiratory, or metabolic diseases.

Overall function was scored in accordance with the subjective functional evaluation of the Musculoskeletal Tumor Society (MSTS) [22]. MSTS scores are assigned on a scale of 0 to 5 in each of 6 categories, with a possible total score range of 0 to 30.

### 2.2. Equipment

Gait analysis was performed using an eight-camera, three-dimensional motion analysis system (Motion Analysis Corp., Santa Rosa, CA, USA; 120 Hz) with two force plates (9260AA6; Kistler Instrumente AG, Winterthur, Switzerland). All participants walked barefoot on a 7 m walkway >3 times, and kinematic data were recorded. We collected and averaged data for >3 successful trials (defined as foot contact achieved on various force plates). Markers were placed on body landmarks according to the Helen Hayes marker set [23].

### 2.3. Data Collection and Processing

Joint kinematics and kinetics were assessed using an inverse dynamic analysis with EVaRT software (ver. 5.0.5; Motion Analysis Corp., Santa Rosa, CA, USA). All data were processed using OrthoTrak software (ver. 6.6.4, Motion Analysis Corp., Santa Rosa, CA, USA) and Microsoft Excel (2016, Microsoft Corp., Redmond, WA, USA). Joint power was calculated by multiplying the joint angular velocities and joint moments in the sagittal plane.

### 2.4. Statistical Analysis

The study parameters are listed in Table 2 [5]. The ground reaction forces (GRFs), joint angles, reaction joint moments, and joint powers were averaged for each of the three groups. Comparisons among the three groups were performed using the Kruskal-Wallis test. Post-hoc inferential analysis (Bonferroni’s test) was used to identify specific groups where significant differential expression occurred. The partial eta squared (η^2^) statistics were used to evaluate effect size. Stevens characterized η^2^ = 0.01 as corresponding to a small effect size, η^2^ = 0.06 to a medium effect size, and η^2^ = 0.14 to a large effect size [24]. All graphics were generated using R. All statistical analyses were conducted using SPSS software (version 23.0, SPSS; Chicago, IL, USA). A *p*-value <0.05 was considered statistically significant.

## 3. Results

### 3.1. Patient Characteristics

The characteristics of the DFG and PTG are listed in Table 3. The resected bone length was greater in the DFG than in the PTG (*p* = 0.002). All patients could walk without an assistive device. The 18 control participants were all male and had a mean age of 32.33 years (range, 26–38). The spatiotemporal gait parameters are listed in Table 4. The DFG and PTG walked with significantly lower velocity than the control group (*p* = 0.040 and *p* = 0.001, respectively), even after normalization of walking velocity correcting for lower limb length. Furthermore, cadence was lower in both the DFG and PTG (*p* = 0.019 and *p* < 0.001, respectively). The step length was not significantly different among the three groups; however, after normalization of step length, that of the DFG and PTG was shorter than that of controls. Meanwhile, the stance and swing phases were not significantly different among the three groups. MSTS score was not significantly different between the two treatment groups (*p* = 0.32).

### 3.2. Comparisons among the Three Groups

#### 3.2.1. Ground Reaction Forces

**Figure 1:**
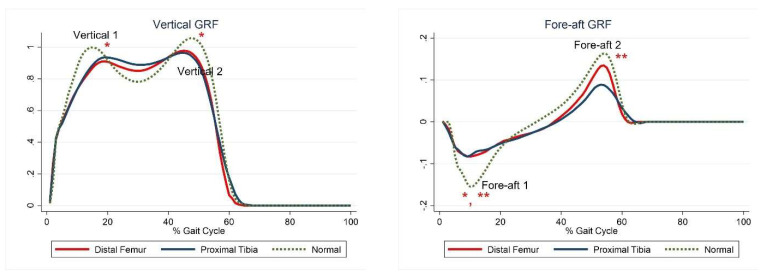
Ground reaction forces. The red lines represent mean values for the distal femur group. The blue lines represent mean values for the proximal tibia group. The dashed lines represent the healthy subjects. * Statistically significant for the comparison between distal femur group and healthy subjects. ** Statistically significant for the comparison between proximal tibia group and healthy subjects.

The first and second peaks of the vertical GRF and the first peak of the fore-aft GRF were significantly lower in the DFG than in the control group, whereas only the first and second peaks of the fore-aft GRF were significantly lower in the PTG than in the control group (Figure 1, Table 5).

#### 3.2.2. Joint Kinematics

**Figure 2:**
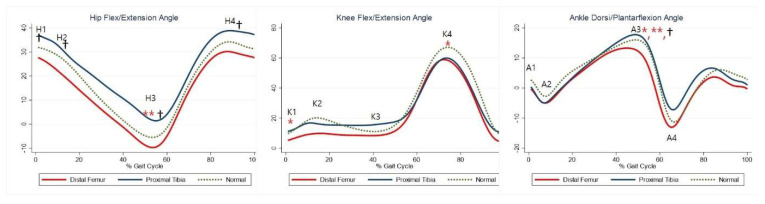
Gait kinematics for each group. The red lines represent mean values for the distal femur group. The blue lines represent mean values for the proximal tibia group. The dashed lines represent the healthy subjects. * Statistically significant for the comparison between distal femur group and healthy subjects. ** Statistically significant for the comparison between proximal tibia group and healthy subjects. † Statistically significant for the comparison between distal femur group and proximal tibia group.

Although not statistically significant, the DFG showed a tendency for greater hip extension than the control group, whereas the PTG showed a tendency for greater hip flexion. Maximal hip extension was significantly greater in the PTG than in the control group (Table 5, H3). The DFG showed a tendency for greater knee extension than the control group (Table 5, K1–2, K4), whereas there was no significant difference in knee joints between the PTG and control group. The maximal dorsiflexion during stance was significantly lower in the DFG and higher in the PTG than in the control group (Table 5, A3). The joint kinematics between the three groups are shown in Figure 2.

#### 3.2.3. Joint Moments and Powers

**Figure 3:**
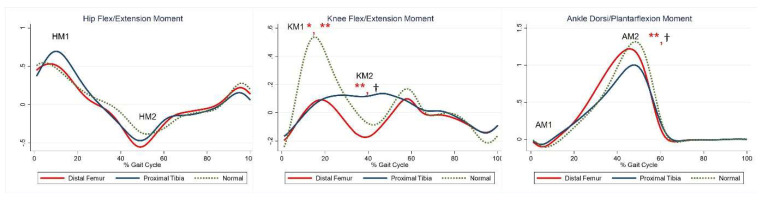
Moment for each group. The red lines represent mean values for the distal femur group. The blue lines represent mean values for the proximal tibia group. The dashed lines represent the healthy subjects. * Statistically significant for the comparison between distal femur group and healthy subjects. ** Statistically significant for the comparison between proximal tibia group and healthy subjects. † Statistically significant for the comparison between distal femur group and proximal tibia group.

**Figure 4:**
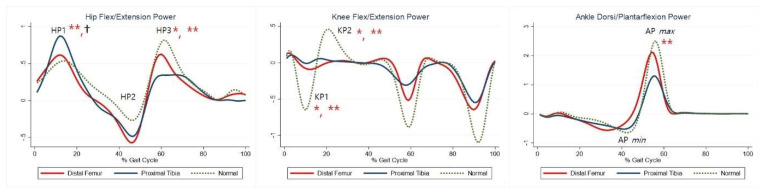
Powers for each group. The red lines represent mean values for the distal femur group. The blue lines represent mean values for the proximal tibia group. The dashed lines represent the healthy subjects. * Statistically significant for the comparison between distal femur group and healthy subjects. ** Statistically significant for the comparison between proximal tibia group and healthy subjects. † Statistically significant for the comparison between distal femur group and proximal tibia group.

The maximal knee extension moment during early stance was lower in the DFG and PTG than in the control group (Table 5, KM). The maximal knee flexor moment during midstance was higher in the PTG than in the control group (Table 5, KM2). The maximal plantar flexion moment was lower in the PTG than in the control group (Table 5, AM2). Compared with the control group, the PTG showed higher power generation at the hip during early stance, and both the DFG and PTG showed lower power generation in late swing (Table 5, HP1, HP3). Further, absorption at the knee and power generation during pre-swing were lesser in the DFG and PTG (Table 5, KP1–2). The PTG tended to show lower power at the ankle during terminal stance (Table 5, AP*max*). Joint moments and powers are shown in Figure 3 and Figure 4, respectively.

### 3.3. Distal Femur Group vs. Proximal Tibia Group

#### 3.3.1. Ground Reaction Forces

There were no significant differences between the two groups.

#### 3.3.2. Joint Kinematics

The DFG showed a tendency for greater hip extension during the gait cycle than the PTG (Table 5, H1–4), whereas there were no significant between-group differences in joint kinematics at the knee. At the ankle, maximal dorsiflexion during stance was lower in the DFG than in the PTG (Table 5, A3).

#### 3.3.3. Joint Moments and Powers

Maximal knee flexor moment during midstance was higher in the PTG than in the DFG (Table 5, KM2). Maximal plantarflexion moment was lower in the PTG than in the DFG (Table 5, AM2). Power generation at the hip during early stance was higher in the PTG than in the DFG (Table 5, HP1). At the ankle, power absorption was lower in the DFG than in the PTG (Table 5, APmin).

## 4. Discussion

Gait analysis according to tumor location (distal femur or proximal tibia) among patients who have undergone knee endoprosthetic replacement is scarce. In most previous studies, the gait patterns of patients who had undergone knee endoprosthesis placement were compared with those of healthy participants, or non-involved sites without consideration of the tumor location [2,5,16,20,25]. Further, although there have been studies on the gait of patients undergoing knee replacement after distal femoral tumor resection, it was not compared with that of patients with proximal tibial tumors [20,21,26]. In this study, we divided patients into two groups—DFG and PTG—according to tumor location, conducted between-group comparisons and comparisons with a healthy control group, and ultimately attempted to explain the difference in gait pattern between the DFG and PTG. This helped us to estimate the potential postoperative walking problems according to tumor location.

### 4.1. Distal Femur Group vs. Control Group

Knee gait parameters were more affected in the DFG than in the control group, with lower knee flexion during early stance in the DFG. A high degree of knee extension during loading response was previously described in patients with distal femoral endoprostheses [17,20,26,27,28]. Mechanically, full knee extension provides stability during loading, as this position is associated with a greater amount of passive stability due to the anterior position of the GRF vector [16]. The high degree of knee extension in the DFG can be explained by the loss of quadriceps strength. Knee flexion in the initial contact and loading response is avoided to protect the weak quadriceps from the strain of decelerating a rapidly flexing knee, thus resulting in a reduced knee extension moment. Therefore, maximal knee flexion in the swing phase was significantly lower in the DFG than in the control group.

Consistent with previous findings [26,29], the DFG demonstrated reduced knee power absorption during pre- and terminal swing. This finding indicates the need for strategies aimed at positioning the GRF vector close to (or even anterior to) the knee joint throughout the loading response. Additionally, compensation for quadriceps weakness was observed in the hip.

Although there was no statistically significant difference compared to healthy participants, we found a shift in the flexion–extension pattern of the hip towards more extension during the entire gait cycle. Further, Rompen et al. [20] described a shifted flexion–extension pattern of the hip. Increasing hip extension prevents the GRF from passing behind the knee joint, due to weakness of the quadriceps, providing an explanation for the observed reduced vertical GRF. Power generation at the hip during terminal stance was significantly reduced in the DFG, suggesting weakness in the hip flexors as they contracted concentrically to lift the lower limb following toe off [16]. This explains the observed reduction in anterior shear GRF. Moreover, a reduced maximal dorsiflexion during stance was observed in the ankle. The reduced dorsiflexion might be due to contraction of the calf muscles to stabilize the tibia as compensation for quadriceps weakness.

### 4.2. Proximal Tibia Group vs. Control Group

Maximal dorsiflexion during stance and maximal ankle power generation were lower in the PTG than in the control group, suggesting that calf muscle weakness could be possibly due to injury during proximal tibial resection [25]. This explains the flexed knee gait observed, although the difference was not statistically significant. The lack of sufficient plantar flexor muscle strength allows the tibia to fall forward as the body vector advances. As such, the tibia advances faster than the femur, causing continued knee flexion [30]. A reduced range of knee flexion causes a reduced knee extensor moment, which affects knee power absorption and generation.

A reduced fore-aft GRF was observed as well. Carty et al. [16] explained that the reduced propulsive force applied to the ground was due to the decrease in rectus femoris power absorption, which may have interrupted the energy transfer along the kinetic chain from knee to foot. It is possible that disruption of the extensor mechanism in PTG patients hinders the energy transfer from the knee to the ankle [12,31]. Further, we noted a reduced plantarflexion moment. In the PTG, in addition to quadriceps weakness, this mechanism is reflected as the effect on calf muscle weakness.

### 4.3. Distal Femur Group vs. Proximal Tibia Group

Gait differed between the DFG and the PTG. Significant between-group differences were observed for the hip. The PTG showed greater hip flexion during the entire gait cycle than the DFG. Hip extension in the DFG can be explained by quadriceps weakness; however, the mechanism for hip flexion in the PTG is unclear. Given that hip extension is used to compensate for quadriceps weakness, positioning the GRF as close to the knee as possible reduces quadriceps demand [5,16,20]. Hip flexion can result from hip extensor weakness. Weakness in hip extensors in patients who underwent endoprosthetic knee replacement, and reported previously, may be associated with weaker ipsilateral body support during early stance [5]. Beebe et al. [32] suggested that hip weakness could be a result of destabilization and emphasized strengthening exercises of the hip extensor muscles. This is possible because calf muscle weakness affects tibia stabilization and, in addition to quadriceps weakness, may cause destabilization in the PTG. Additionally, the disruption of the extensor mechanism caused by detachment of the patellar tendon, required for proximal tibia resection, might explain this. However, hip muscle and calf muscle strength was not measured. Further studies are needed to confirm hip extensor weakness.

The maximal knee flexor moment during midstance was higher in the PTG than in the DFG or in the control group (Table 5, KM2). This can be explained by the flexed knee gait pattern in the PTG, even if the difference was not statistically significant. However, given the small number of patients, statistical errors cannot be excluded. Additionally, considering that the knee flexion moment in the midstance region is affected by gastrocnemius activity, further studies on gastrocnemius strength or firing are warranted.

Maximal dorsiflexion during stance was higher in the PTG than in the DFG, even when compared to healthy participants. Maximal power absorption was higher in the PTG than in the DFG, and these findings support the more pronounced calf muscle weakness in the PTG than in the DFG.

The extent of bone resection was significantly higher in the DFG than in the PTG. Carty et al. [16] reported that the amount of soft tissue removal was the most predictive factor of locomotor function. However, in their study, resected bone length was not significantly related with kinetic and kinematic values. In our study, despite the difference in bone length between the two groups, it was not possible to quantitatively estimate the amount of soft tissue removal, and, particularly, extensor strength could not be measured. Further studies are needed on the relationship between resected bone length and soft tissue removal and their effect on gait.

A successful reconstruction of the extensor mechanism to the proximal tibia is key to a good functional outcome for the PTG [33]. Although there is no standard method of achieving the extensor mechanism, a number of techniques have been used to reconstruct the patellar tendon [7,34,35,36,37,38,39,40]. Pilge et al. [41] reported that there was almost no extensor lag when the patellar tendon was reconstructed using the Kosa cord method, and that reducing the extensor lag was crucial. Jentzsch et al. [42] revealed that, after reconstruction using medial gastrocnemius flap, the patellar tendon can be stretched up to 2 years after surgery, and that patella alta is associated with a lower MSTS score. In our patients, the patellar tendon was directly fixed to the prosthesis using a nonabsorbable suture and the medial gastrocnemius muscle was reattached to the retinacula and pes anserinus. We believe that stabilizing the patellar tendon and restoring the extensor mechanism, by maintaining an appropriate length, affects the knee’s range of motion, which affects gait.

The rehabilitation process following an endoprosthetic reconstruction around the knee can differ according to the resected muscle. Moreover, the rehabilitation of proximal tibial replacement is the opposite of that following a distal femoral replacement, or total knee joint replacements in general [43]. Regaining extensor function following patellar tendon reconstruction in the PTG is critical [12,31,43]. Therefore, the recovery of full extension is a more relevant issue in early phases of rehabilitation for the PTG than for the DFG.

### 4.4. Limitations

Our study had several limitations. Firstly, the sample size was small (DFG = 7, PTG = 9, and healthy participants = 18), preventing us from guaranteeing statistical power in each comparison and allowing discussion of only the detected differences. Secondly, there may have been selection bias, as the patient population achieved good functional outcomes (they could walk without an assistive device). However, we attempted to reduce the influence of confounding factors of gait parameters by excluding patients who had undergone revision surgery or had deformities. We acknowledge that the gait assessment time from surgery seems to be too varied between subjects. However, we included patients who had undergone surgery ≥2 years prior, which was expected to reduce the effect of surgery itself on gait analysis. Finally, we lacked information on muscle activities or electromyography and on the strength of the hip extensors and plantar flexors, which would have helped to deepen the discussion. Further analysis, including musculoskeletal stimulation and functional outcomes, is required to verify the significant changes in gait.

## 5. Conclusions

Gait pattern differed according to tumor location in patients who had undergone knee endoprosthesis after tumor resection. There were changes in knee, hip, and ankle parameters due to quadriceps weakness in the DFG and changes in ankle parameters in the PTG. Further, there were differences in hip parameters between the DFG and PTG. Thus, rehabilitation strategies need to be patterned according to tumor location.

## Figures and Tables

**Table 1:** Patient characteristics at the time of measurement.

Patient	Sex	Age at Operation (Year)	Diagnosis	Stage	Site	Tumor Size (cm)	Resected Muscles	Assessment Time from Surgery (Year)
1	M	15.08	Osteosarcoma	IIb	Distal femur	11 × 6.5 × 4.5	VI, VL	3.2
2	M	16.42	Osteosarcoma	IIb	Distal femur	9 × 6.5 × 6.5	VI	2.1
3	M	18.58	Osteosarcoma	IIb	Distal femur	17.8 × 8.2 × 4.5	VM, VI, partial VL, GC medial & lateral head	9.6
4	M	15.17	Osteosarcoma	IIb	Distal femur	12.4 × 5.9 × 5.4	VI, VL	3.1
5	F	17.67	Osteosarcoma	IIb	Distal femur	14 × 7 × 6	VM, VI, GC medial head	3.2
6	M	13.33	Osteosarcoma	IIb	Distal femur	6.6 × 6.6 × 4.5	RF, VM, VI, VL	2.3
7	M	19.08	Ewing’s sarcoma	IIb	Distal femur	10 × 5.5 × 5.5	RF, VM, VI, VL	11.8
8	M	49.58	Giant Cell Tumor	IIb	Proximal tibia	7.5 × 5.2 × 4.1	GC medial head	5.0
9	F	30.17	Osteosarcoma	III	Proximal tibia	8 × 7 × 4.5	GC medial head	2.0
10	F	17.42	Osteosarcoma	III	Proximal tibia	3.1 × 5.2 × 3.1	TA, TP, extensor hallucis & digitorum, GC lateral head, peroneal nerve	4.0
11	F	17.25	Osteosarcoma	III	Proximal tibia	6 × 3 × 3	GC medial head	2.0
12	M	14.50	Osteosarcoma	IIb	Proximal tibia	8.6 × 6.0 × 5.5	Popliteus, partial flexor digitorum, partial TP GC medial head	14.0
13	F	16.33	Osteosarcoma	IIb	Proximal tibia	2.5 × 4.7 × 2.6	Flexor digitorum longus, soleus, popliteus, GC medial head	10.0
14	F	45.58	Giant Cell Tumor	IIb	Proximal tibia	4.5 × 4.0 × 2.5	GC medial head	9.0
15	M	16.08	Osteosarcoma	IIb	Proximal tibia	10 × 2.2 × 3.5	Popliteus, GC medial head	2.0
16	M	31.50	Osteosarcoma	IIb	Proximal tibia	13 × 10 × 7	GC medial head, flexor digitorum longus	2.0

VI, vastus intermedius; VM, vastus medius; VL, vastus lateralis; GC, gastrocnemius; RF, rectus femoris; TA, tibialis anterior; TP, tibialis posterior.

**Table 2:** Kinematic, kinetic, and energetic gait parameters of interest.

Name	Description
Ground Reaction Forces, % BW	
GRF Fore-aft 1	Maximal aft force
GRF Fore-aft 2	Maximal fore force
GRF Vertical 1	Maximal vertical force during early stance
GRF Vertical 2	Maximal vertical force during late stance
(reaction) Joint Moments, Nm/(kg·m)	
HM1	Maximal hip extension moment during stance
HM2	Maximal hip flexion moment during stance
KM1	Maximal knee extension moment during early stance
KM2	Maximal knee flexion moment during mid stance
AM1	Maximal dorsiflexion moment during stance
AM2	Maximal plantarflexion moment
Joint Angles	
H1	Hip flexion at initial contact
H2	Maximal hip flexion during early stance
H3	Maximal hip extension
H4	Maximal hip flexion during swing
K1	Knee flexion at initial contact
K2	Maximal knee flexion during early stance
K3	Knee flexion at toe off
K4	Maximal knee flexion during late stance
A1	Ankle dorsiflexion at initial contact
A2	Maximal plantarflexion during early stance
A3	Maximal dorsiflexion during stance
A4	Ankle plantarflexion at toe off
Joint Powers, W/(kg·m)	
HP1	Maximal hip joint power during early stance
HP2	Minimal hip joint power during late stance
HP3	Maximal hip joint power during swing
KP1	Minimal knee joint power during early stance
KP2	Maximal knee joint power during early stance
AP*min*	Minimal ankle joint power
AP*mean*	Mean negative ankle power during stance
AP*max*	Maximal ankle joint power

**Table 3:** Comparison between distal femur group and proximal tibia group.

	Distal Femur Group (*n* = 7)	Proximal Tibia Group (*n* = 9)	*p*-Value ^a^
Age at operation, years Median (range)	17.1 (15.1–19.1)	26.5 (16.0–49.6)	0.252
Body mass index (kg/m^2^) Median (range)	21.7 (17.3–22.3)	18.0 (15.5–26.2)	0.681
Assessment time from surgery, years Median (range)	7.1 (2.0–11.8)	4.0 (2.0–13.7)	0.423
Resected bone length (mm) Median (range)	162.6 (146.2–259.5)	130.7 (108.2–183.7)	0.002
MSTS score, (mean ± SD)	23 ± 3.24	24.4 ± 2.07	0.32

MSTS, Musculoskeletal Tumor Society; statistically significant values in bold ^a^; Mann–Whitney test.

**Table 4:** Comparison of spatiotemporal gait parameters between patients and healthy participants.

	Distal Femur (*n* = 7), Mean	Proximal Tibia (*n* = 9), Mean	Healthy Participants (*n* = 18), Mean	*p*-Value ^a^		
				DFG vs. Healthy	PTG vs. Healthy	DFG vs. PTG
Stance phase (%cycle)	59.39	60.68	60.68	0.124	1.000	0.246
Swing phase (%cycle)	40.61	39.32	39.32	0.124	1.000	0.246
Double support (%cycle)	10.33	11.27	10.41	1.000	1.000	1.000
Velocity (cm/s)	107.13	95.30	118.18	0.040	0.001	0.686
Normalized velocity ^b^	0.13	0.12	0.15	<0.001	0.001	0.303
Stride length (cm)	117.82	110.38	122.16	0.795	0.036	0.305
Normalized stride length ^b^	0.14	0.14	0.15	0.004	0.038	0.500
Cadence (step/min)	108.85	102.92	115.72	0.019	<0.001	0.630
Normalized cadence ^b^	0.13	0.13	0.14	0.002	0.018	0.268
Step length (cm)	59.44	56.21	61.05	0.720	0.071	0.490
Normalized step length ^b^	0.07	0.07	0.08	0.012	0.042	0.379

SD, standard deviation; DFG, distal femur group; PTG, proximal tibia group (statistically significant values in bold); ^a^ Kruskal–Wallis test; ^b^ normalization values corrected for lower limb length.

**Table 5:** Comparison of kinetic and kinematic parameters between patients and healthy participants.

	Distal Femur (*n* = 7), Mean (SD)	Proximal Tibia (*n* = 9), Mean (SD)	Healthy Participants (*n* = 18), Mean (SD)	*p*-Value ^a^			Effect Size ^b^ (η^2^)
				DFG vs. Healthy	PTG vs. Healthy	DFG vs. PTG	
*Ground Reaction Forces*							
GRF Vertical 1	0.91 (0.10)	0.97 (0.14)	1.04 (0.11)	0.034	0.174	0.683	0.131(M)
GRF Vertical 2	0.99 (0.10)	0.98 (0.13)	1.07 (0.05)	0.018	0.087	0.941	0.205(L)
GRF Fore-aft 1	−0.09 (0.03)	−0.09 (0.03)	−0.16 (0.03)	<0.001	<0.001	1.000	0.641(L)
GRF Fore-aft 2	0.14 (0.04)	0.10 (0.05)	0.17 (0.02)	0.256	<0.001	0.133	0.493(L)
Joint Angles							
H1	27.64 (6.49)	36.98 (8.60)	31.88 (5.74)	0.146	0.239	0.014	0.155(L)
H2	27.65 (6.51)	36.98 (8.60)	31.93 (5.81)	0.142	0.258	0.015	0.151(L)
H3	−9.88 (5.94)	0.98 (10.05)	−5.63 (6.21)	0.188	0.044	0.013	0.157(L)
H4	30.71 (5.13)	39.22 (7.67)	34.25 (4.99)	0.208	0.216	0.019	0.136(M)
K1	5.22 (6.65)	11.22 (6.61)	9.77 (3.55)	0.035	0.808	0.040	0.109(M)
K2	10.27 (7.37)	16.50 (7.21)	19.05 (8.50)	0.034	0.551	0.297	0.105(M)
K3	7.80 (7.65)	14.99 (8.50)	10.56 (3.34)	0.586	0.199	0.072	0.070(M)
K4	59.55 (8.23)	60.08 (10.05)	67.41 (3.25)	0.006	0.062	0.686	0.271(L)
A1	−0.30 (3.60)	0.40 (3.05)	2.74 (3.31)	0.110	0.139	1.000	0.085(M)
A2	−5.23 (3.29)	−5.79 (2.66)	−2.86 (4.09)	0.367	0.120	1.000	0.048(S)
A3	13.48 (3.00)	18.93 (2.10)	16.21 (2.48)	0.073	0.038	<0.001	0.349(L)
A4	−14.15 (7.38)	−7.62 (15.35)	−11.86 (3.80)	1.000	0.271	0.217	0.018(S)
Joint Moments, Nm/(kg·m)							
HM1	0.57 (0.12)	0.72 (0.25)	0.57 (0.13)	0.814	0.828	0.462	−0.031(S)
HM2	−0.56 (0.32)	−0.51 (0.32)	−0.40 (0.11)	0.510	0.891	1.000	−0.033(S)
KM1	0.12 (0.22)	0.16 (0.19)	0.55 (0.30)	0.009	0.006	1.000	0.328(L)
KM2	−0.19 (0.12)	0.10 (0.20)	−0.64 (0.18)	0.064	0.017	0.012	0.349(L)
AM1	−0.11 (0.07)	−0.09 (0.10)	−0.11 (0.049)	1.000	0.381	0.468	−0.015(S)
AM2	1.24 (0.15)	1.05 (0.14)	1.33 (0.07)	0.251	<0.001	0.019	0.484(L)
Joint Powers, W/(kg·m)							
HP1	0.66 (0.17)	0.98 (0.30)	0.680 (0.17)	1.000	0.014	0.034	0.188(L)
HP2	−0.61 (0.36)	−0.55 (0.45)	−0.30 (0.09)	0.058	0.487	0.456	0.078(M)
HP3	0.65 (0.20)	0.49 (0.10)	0.86 (0.14)	0.008	<0.001	0.141	0.561(L)
KP1	−0.13 (0.13)	−0.05 (0.08)	−0.67 (0.62)	0.002	<0.001	0.429	0.345(L)
KP2	0.07 (0.06)	0.08 (0.11)	0.49 (0.37)	0.006	0.002	1.000	0.400(L)
AP*min*	−0.62 (0.14)	−0.80 (0.18)	−0.74 (0.23)	0.215	0.905	0.045	0.038(S)
AP*mean*	−0.27 (0.08)	−0.32 (0.10)	−0.24 (0.05)	0.577	0.169	0.909	0.022(S)
AP*max*	2.23 (0.60)	1.69 (0.70)	2.66 (0.37)	0.272	0.002	0.245	0.266(L)

SD, standard deviation; DFG, distal femur group; PTG, proximal tibia group; S, small; M, moderate; L, large; statistically significant values in bold. ^a^ Kruskal–Wallis test ^b^ partial eta squared test.

## Data Availability

Deidentified individual participant data (including data dictionaries) will be made available, in addition to study protocols, the statistical analysis plan, and the informed consent form. The data will be made available upon publication to researchers who provide a methodologically sound proposal for use in achieving the goals of the approved proposal. Proposals should be submitted to stjung@jnu.ac.kr.
